# A Proposed New Index for Clinical Evaluation of Interproximal Soft Tissues: The Interdental Pressure Index

**DOI:** 10.1155/2014/345075

**Published:** 2014-04-01

**Authors:** Checchi Luigi, Montevecchi Marco, Marucci Gianluca, Checchi Vittorio

**Affiliations:** ^1^Department of Periodontology & Implantology, DIBINEM, Alma Mater Studiorum-University of Bologna, Via San Vitale 59, 40100 Bologna, Italy; ^2^Section of Pathology, Department of Haematology and Oncological Sciences, Bellaria Hospital, University of Bologna, Via Altura 3, 40100 Bologna, Italy; ^3^Department of Medical Sciences, University of Trieste, Piazza dell'Ospitale 1, 34100 Trieste, Italy

## Abstract

The interdental pressure index (IPI) is introduced to specifically evaluate clinical interproximal-tissue conditions and assess the effect of interproximal hygiene stimulation. This index scores clinical responses of periodontal tissues to the apical pressure of a horizontally placed periodontal probe. It is negative when gingival tissues are firm, bleeding-free, and slightly ischemic by the stimulation; otherwise it is positive. The clinical validation showed high intraoperator agreement (0.92; 95% CI: 0.82–0.96; *P* = 0.0001) and excellent interoperator agreement (0.76; 95% CI: 0.14–1.38; *P* = 0.02). High internal consistency with bleeding on probing (*κ* = 0.88) and gingival index (Cronbach's *α* = 0.81) was obtained. Histological validation obtained high sensitivity (100%) and specificity (80%) for IPI+ toward inflammatory active form. The same results were recorded for IPI− toward chronic inactive form. IPI results as a simple and noninvasive method with low error probability and good reflection of histological condition that can be applied for oral hygiene motivation. Patient compliance to oral hygiene instructions is essential in periodontal therapy and IPI index can be a practical and intuitive tool to check and reinforce this important aspect.

## 1. Introduction

Many clinical indexes, instrumental examinations, and laboratory tests are today available in order to study and define periodontal condition. Among them, clinical indexes remain the most commonly used criteria for an ordinary evaluation, due to their viability. Periodontal soft-tissue responses to nonsurgical periodontal therapy commonly show general improvement after treatment on all conventionally used clinical indexes [1, 2]. This positive response is an obvious benefit of professional instrumentation and a consequence of improved patient oral hygiene habits [3].

Interdental papilla or, more generally, soft tissues of the interproximal areas is mostly affected by a correct soft tissue stimulation. Cantor and Stahl [4] and Checchi et al. [5] demonstrated that daily use of an interproximal hygiene device, adjacent to a periodontal pocket, results in keratinization of the dental col and shrinkage and flattening of the papillae, with a consequent decrease in pocket depth.

Despite the perceptible advantages of well-stimulated interproximal tissues [6, 7], very few indexes have tried to specifically measure these factors. Our review of the literature found only few indexes created to evaluate papilla inflammation [8–10].

In 1975, Saxer and Mühlemann introduced the Papillary Bleeding Index (PBI) [8], a four-grade index based on both the extent of bleeding and the time it takes for bleeding to occur after stimulation with a periodontal probe. In 1984, the dichotomous Eastman Interdental Bleeding Index (EIBI) was proposed [9]. The fourfold horizontal insertion of a wooden interdental cleaner was used to stimulate the interproximal gingival tissues and the presence or absence of bleeding within 15 seconds was recorded. In 1989, Graves and colleagues, comparing the clinical effectiveness of different hygienic tools in reducing interproximal bleeding sites, stated that the Interdental Bleeding Index method, compared to probing, is a simplified way to assess interproximal gingival inflammation [10].

Unfortunately, in our opinion, these indexes lack viability and neglect important clinical signs other than bleeding. The aim of this study is to introduce the “Interdental pressure index” (IPI), a new index developed specifically to record interproximal-tissue status.

## 2. Materials and Methods

### 2.1. Scoring Method

**Figure 1:**
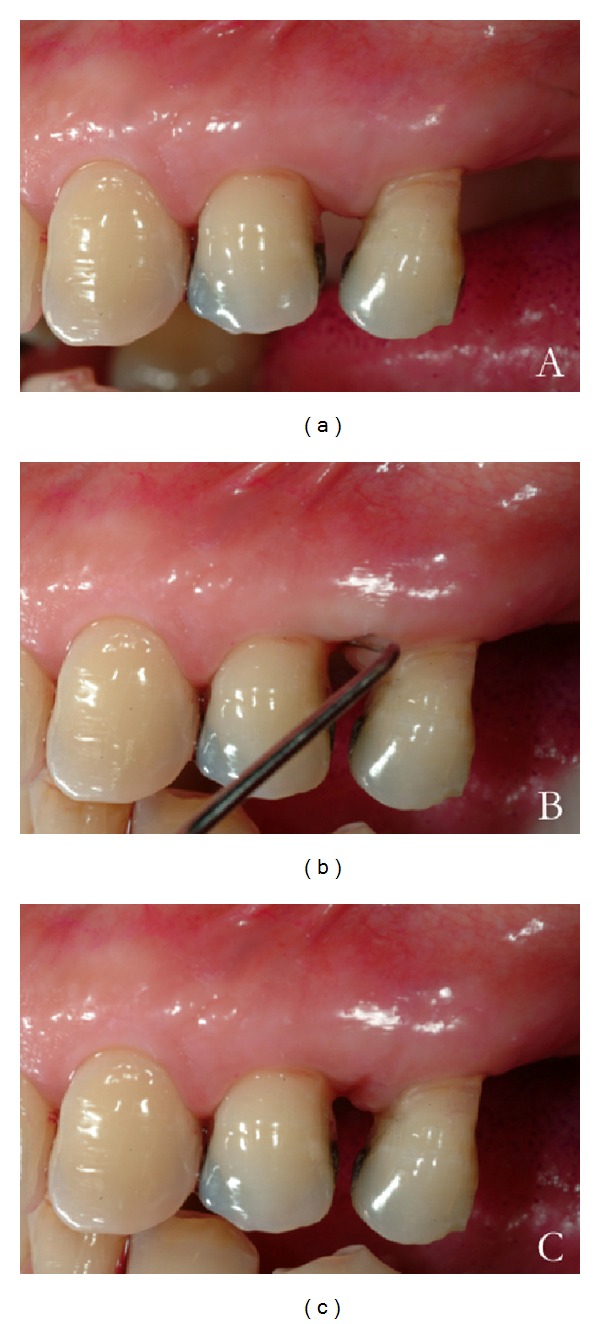
Example of interdental pressure index (IPI) positive score. (a) Tissues are inflamed and not firm. (b) Compression of the tissue shows its mobility, inducing an ischemic response. (c) After compression, the papilla returns slowly to its original position.

**Figure 2:**
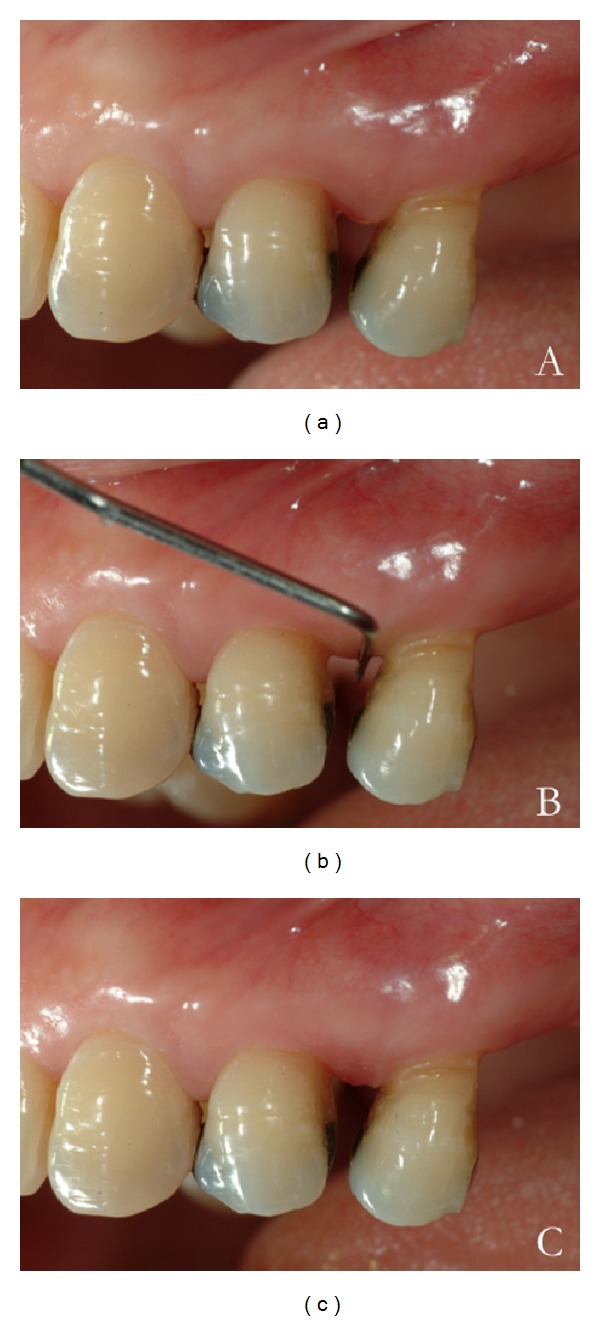
Example of interdental pressure index (IPI) negative score. (a) The tissues are firm and the col is keratinized. (b) Compression of the tissue shows a firm and tonic papilla. (c) Absent or a slight ischemic response is induced.

The IPI scoring system is based on a positive or negative assignment, representing tissue reaction to apical pressure in the interdental area, applied by a horizontally placed periodontal probe (Figures 1 and 2). The pressure applied by the probe should be firm and continuous until reaching maximum compression with minimal discomfort to the patient.

IPI is dedicated to periodontally affected areas and is assessable both buccally and from the lingual/palatal side. The index is negative when the gingival tissues result firm, slightly ischemic, and not bleeding. In contrast, the index is positive when the gingival tissues are spongy, dimensionally unstable with a consistent color variation. A bleeding response can be also induced. To assign a positive score, at least one of the previously mentioned clinical signs is needed (Table 1).

### 2.2. Clinical Validation

**Figure 3:**
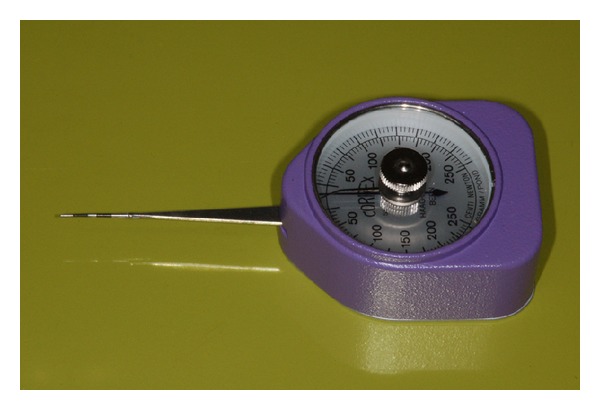
Calibrated dynamometer modified by sticking a periodontal probe tip to the terminal portion of the sensory arm.

**Figure 4:**
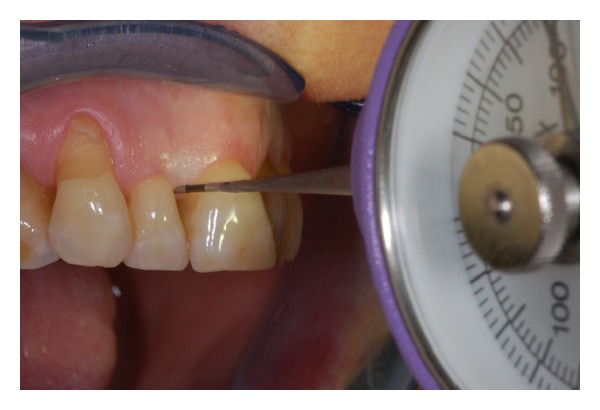
Clinical application of the dynamometer while studying the IPI recording force.

In order to define the entity of forces for IPI scoring, a clinical evaluation, by means of a specifically modified calibrated dynamometer (Correx, Haag-Streit AG, Koniz, Switzerland) (Figures 3 and 4), was performed in 2007 on 40 consecutive periodontal patients of the Department of Periodontology and Implantology of University of Bologna (Italy). A Visual Analogical Scale (VAS) was used to estimate the patient discomfort perception correlated to interproximal pressure (0 = no pain, 10 = unbearable pain). The force ranged between 100 and 200 cN and the 80% of subjects assigned a VAS score ≤3. The association between low values of force and low VAS scores was statistically significant (Spearman's RHO = 0.7, *P* = 0.001). From this observation it can be assumed that a correct IPI recording force should induce a minimal patient discomfort.

Because the aim of this pilot study was to introduce IPI, no predefined sample size calculation was performed.

To clinically validate IPI, one sample of 25 interproximal areas, one site from each patient, was examined. Subjects were selected in 2008 from consecutive periodontal patients under active treatment at the Department of Periodontology and Implantology of Bologna University, according to the following inclusion criteria: diagnosis of chronic periodontitis [11], American Society of Anesthesiologists (ASA) status 1 [12], and at least a complete dentate quadrant (except the third molar). Smokers were excluded. Data collection occurred during reevaluation time after 30 days from initial preparation. Informed consent was obtained from all participants. Tenets of the Declaration of Helsinki were followed. The study sample was composed of 15 female and 10 male, with a mean age of 56 years (range 45–66).

No clinically significant variability was observed among sites within the same subject, and no trend was observed among the same sites in different subjects. Consequently, lots were drawn using a table of pseudorandom numbers to randomize the selection of the interproximal areas studied.

To evaluate inter- and intraoperator agreement, two examiners (MM, VC), both experienced dentists with graduate training in periodontics, collected IPI, gingival index (GI) [13], and pocket bleeding index (PBI) [14]. A standardized periodontal probe (CP11; Hu-Friedy, Chicago, IL, USA) was used for the clinical evaluation. Examiners were blinded to each other and the two observations were taken with an interval of at least 15 minutes. Intraoperator agreements were calculated for both examiners.

Because the four categories of the GI denote a progressive increase in clinically detectable inflammation, we attempted to dichotomize this value to enhance statistical comparison with IPI. From a clinical point of view, it was considered that categories 0 and 1 and categories 2 and 3 could be grouped, providing two GI values. This dichotomization was statistically validated.

### 2.3. Histological Validation

In order to study the histological features related to the index, during osseous resective surgeries performed in 2008, 15 IPI+ and 15 IPI− interproximal gingival biopsy specimens were collected in 2008. Thirty periodontal patients [17 females, 13 males, mean age 58 (range 45–66)] were selected according to the following inclusion criteria: initial diagnosis of chronic periodontitis, ASA status 1 and no smokers. All treatments were performed at the Department of Periodontology and Implantology, University of Bologna. Informed consent was obtained from all participants and the tenets of the Declaration of Helsinki were followed.

Specimens consisted of mucosal fragments with major axes measuring 0.5–1 cm. Tissues were fixed in 10% buffered formalin and embedded in paraffin. Blocks were serially cut, stained with hematoxylin and eosin (H&E) and sirius red and immunostained with anti-CD31 (Clone 1A10, prediluted; Cell Marque, Rocklin, California, USA) and anti-CD34 (Clone QBend/10, prediluted; Ventana, Roche, Basel, Switzerland) antisera to indicate vascular structures. About five histological sections were examined by one of the authors (AM) in each case. A three-class grading system based on the semiquantitative evaluation of five histological parameters (fibrosis, hyperkeratosis, amount of blood vessels, inflammation, epithelial hyperplasia) was used. The fibrosis score, evaluated on sirius red-stained slides, graded the intensity of the fibrosis and the modification of the tissutale structure. The hyperkeratosis score, analysed on H&E-stained slides, was based on the quantity of keratinization. The vascular score was evaluated both on CD31- and CD34-stained slides, considering the number and the alterations of structure of blood vessels. The inflammation score, evaluated on H&E-stained slides, was based on the extent of inflammatory infiltrates. The epithelial hyperplasia score, evaluated on H&E-stained slides, was calculated according to the size (length and width) of rete ridges.

As reported in Table 2, a score ranging from 1 to 3 was assigned to each parameter. When the sum of the five scores was ≤8, the case was assigned to grade I and considered representative of an acute active form of periodontal disease. If the sum was ≥12, the case was assigned to grade III and considered representative of a chronic inactive form of periodontal disease. Finally, cases with a sum of 9–11 were assigned to grade II and considered intermediate forms.

### 2.4. Statistical Analyses

Proportion (%) ± standard error was used for the description of IPI and bleeding on probing (BOP; nominal scale); median and interquartile range were used for the description of GI (ordinal scale). The reliability of IPI was assessed by measuring its reproducibility (intra- and interoperator agreement) using the intraclass correlation coefficient and the kappa statistic, respectively. The internal consistency of IPI was evaluated using Cronbach's alpha coefficient, and its homogeneity was measured using GI and BOP, two widely used periodontal indexes for the clinical identification of soft-tissue inflammatory conditions. Intraoperator agreement for GI and BOP was assessed using the intraclass correlation coefficient. Sensitivity, specificity, and positive and negative predictive values were also computed. Fisher's exact test was used to evaluate the significance of the association between IPI and PBI and GI, and the Chi-square test was used to evaluate the significance of the association between IPI and histological assignments. An alpha value of 0.05 was considered to indicate statistical significance. Data were analyzed using the SPSS software (ver. 13.0; SPSS, Inc., Chicago, IL, USA).

## 3. Results

### 3.1. Reliability Analysis

Among the tested areas, 48 ± 10% were positive for IPI and 56 ± 10% showed bleeding on probing. The median GI value was 1 (interquartile range: 1-2). The intraclass correlation coefficients of the study indexes were calculated for both clinical examiners. Data for GI were 0.98 (95% CI: 0.95–0.99; *P* = 0.0001) and 0.96 (95% CI: 0.91–0.98; *P* = 0.0001). The remaining indexes showed similar values in both examiners, respectively: 0.92 for PBI (95% CI: 0.83–0.97; *P* = 0.0001) and 0.92 for IPI (95% CI: 0.82–0.96; *P* = 0.0001). The kappa statistic was 0.76 (95% CI: 0.14–1.38; *P* = 0.02), indicating excellent interoperator agreement according to Landis and Koch [15]. The internal consistency of IPI with PBI was high (*κ* = 0.88) and the internal consistency of IPI with GI was lower (Cronbach's *α* = 0.73). To improve this internal consistency, validation criteria were calculated using different cutoffs for GI. The best cutoff resulted from grouping the GI values 0-1 and 2-3, as reported in Table 3; the Cronbach's alpha coefficient was then recalculated. With this modification, the internal consistency of IPI with GI increased (Cronbach's *α* = 0.81).

### 3.2. Clinical Validation

The comparison of IPI with PBI and GI values is reported in Table 4 : 100% of the areas positive for IPI showed bleeding on probing (*P* = 0.0001) and 82% of areas positive for IPI were classified as having the highest GI value (*P* = 0.001). Using PBI as a criterion for validation, sensitivity was 79%, specificity was 100%, positive predictive value was 100%, and negative predictive value was 79%. Using the highest GI values (2 or 3) as a criterion for validation, sensitivity was 82%, specificity was 86%, positive predictive value was 82%, and negative predictive value was 86%.

### 3.3. Histological Validation

**Figure 5:**
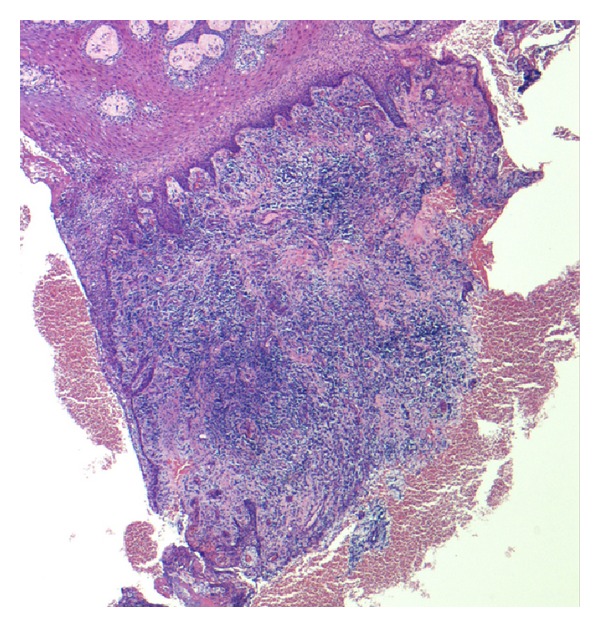
A case scored as grade I (acute active form). This fragment of oral mucosa shows a dense inflammatory infiltrate, a large number of blood vessels, moderate epithelial hyperplasia, mild fibrosis, and inconspicuous hyperkeratosis (EE, ×40 magnification).

**Figure 6:**
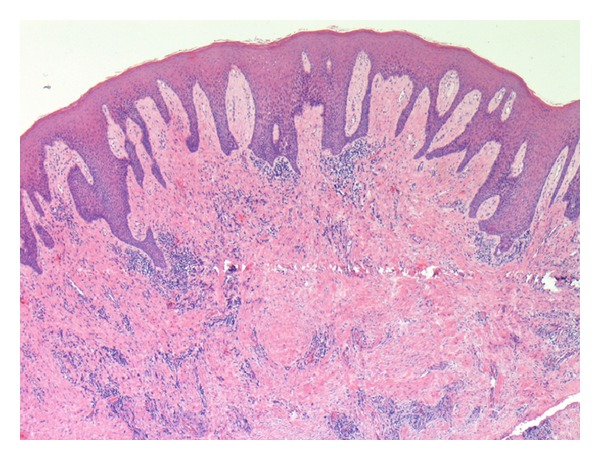
A case scored as grade III (chronic inactive form). This specimen of oral mucosa demonstrates collagen deposition, hyperkeratosis, moderate epithelial hyperplasia, a very mild inflammatory infiltrate, and few blood vessels (EE, ×40 magnification).

**Figure 7:**
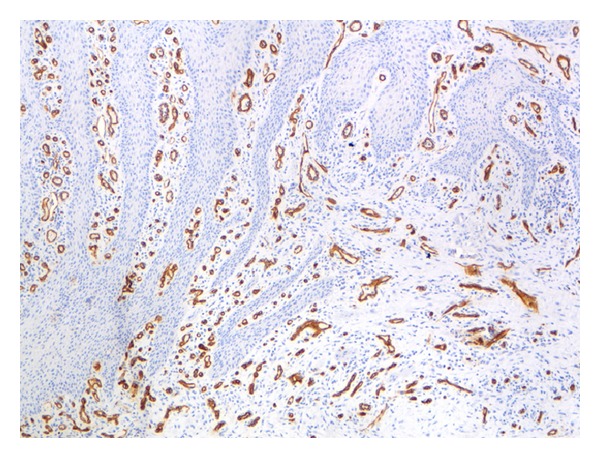
A case scored as grade I (acute active form). This biopsy of oral mucosa shows marked epithelial hyperplasia, a mild-to-moderate inflammatory infiltrate, and a large number of blood vessels immunostained with the anti-CD34 antibody (CD34, ×100 magnification).

**Figure 8:**
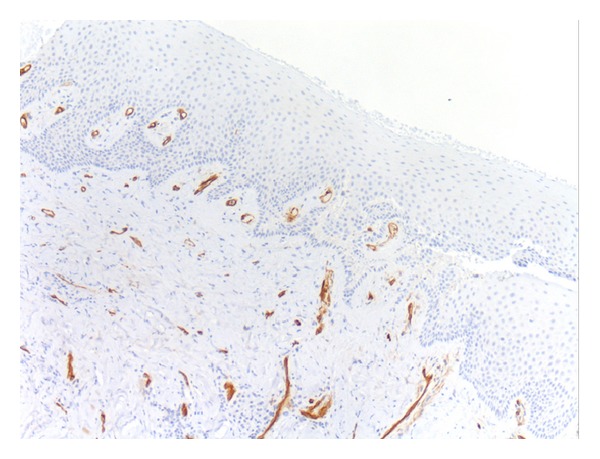
A case scored as grade III (chronic inactive form). This biopsy shows few blood vessels stained with the anti-CD34 antibody, fibrosis, and only focal inflammatory infiltrate (CD34, ×100 magnifications).

The findings of microscopic examinations are reported in Table 5. Twelve cases were classified as grade I (acute active forms; Figure 5) and 12 cases were identified as grade III (chronic inactive forms; Figure 6). Immunostaining with the anti-CD34 antiserum appeared to be more useful and specific than with the anti-CD31 antiserum, because CD34 stains endothelial cells selectively (Figures 7 and 8) and facilitates the recognition of the amount of blood vessels, particularly in cases with a severe inflammatory infiltrate which can obscure the background. Six specimens displayed a grade II pattern (intermediate inflammatory form) and among them, both IPI classes were equally represented. The association between IPI and histological classification was significant (*P* = 0.0001). The sensitivity of IPI+ for acute inflammatory active form was 100%, with a specificity of 80% and a predictive positive value of 80%. The same results for sensitivity, specificity, and predictive positive value were obtained between IPI and the chronic inactive form.

## 4. Discussion

Clinicians and researchers have developed various methods to evaluate oral hygiene conditions and periodontal status in different population groups [16–22]. To arrange the multitude of proposed indexes, Lang and colleagues suggested a classification with five different categories [23], taking their stand from the concept that the purpose of evaluation determines the most appropriate index to use. Self-screening, patient education and motivation, epidemiological surveys, therapeutic purposes, and research have different needs and specific indexes are consequently advisable. IPI has been developed for clinical situations where an easy and rapid evaluation of interproximal soft tissues is required, for instance, during patient oral hygiene motivation. Notwithstanding the multiple signs considered by IPI other than bleeding, a dichotomous outcome was chosen to make it practical. For a detailed periodontal case analysis, more specific and precise diagnostic tests will likely be needed.

Among the periodontal indexes described in the scientific literature, few have focused on interdental soft tissues. To our knowledge, only two clinical indexes, respectively, EIBI and PBI, have been dedicated to this area [8–10]. IPI relies on similar concepts but seeks to increase the feasibility of examination by reducing the interdental stimulus to a single compression and using an ordinary periodontal probe, rather than a wooden interdental cleaner. Furthermore, IPI considers other important clinical signs, like swelling and tissues discoloration, which are neglected by both previous indexes.

The high agreement obtained in the intra- and interexaminer evaluations showed good reproducibility. Notwithstanding the absence of an objective control in the applied pressure and the subjectivity of observation for signs like discoloration and swelling, IPI seems to be effective for clinical use. The high reproducibility obtained could be explained by the previously reported observation that a dichotomous determination seems to have higher reproducibility on repeated testing than a quantitative measurement [20]. The fact that the examiners involved in the validation process are both very familiar with IPI should also be considered.

The considerable homogeneity obtained in the present validation between IPI and two widely accepted indexes (GI and PBI) suggests that IPI could be considered a reliable indicator of clinical soft tissues condition.

Clinical periodontal signs are macroscopic expressions of histological features. Focusing on interproximal tissues, Abrams and colleagues [9] demonstrated a morphometric analysis that bleeding tissues after mechanical stimulation have a significantly greater amount of inflammatory infiltration. In their studies on interproximal hygiene stimulation, Cantor and Stahl [4] and Checchi et al. [5] also observed clear correlations between clinical aspects and epithelial keratinization. The ability of IPI to indicate the periodontal soft tissue condition has been confirmed by a multiparametric histological evaluation. While Abrams et al. [9] considered two histological parameters (fibrosis and inflammatory infiltrate), a three-class grading system was used to examine five histological features in the present study. Such an approach permits more accurate cases classification. For example, 9 of the 30 harvested samples showed a moderate grade of dense collagen deposition/fibrosis (usually more present in chronic forms), but concerning the other parameters, these cases were included in the acute active forms. In particular, evaluation of the degree of hyperkeratosis may contribute to the identification of “chronic” status, whereas the presence of hyperplastic/regenerative epithelium and a significant amount of blood vessels/granulation tissue are reliable markers of “acute” status. Thus, the high sensitivity, specificity, and positive predictive value obtained in the present study confirm the strong correlation between macro- and microscopic findings.

Accepting individual differences, periodontal soft tissues generally reflect patient's oral hygiene conditions [24]. Because tissues need time to complete the modification of clinical aspects, whereas dental plaque is readily removable, the former should be considered a better indicator of patients oral hygiene compliance. This general consideration is particularly appropriate for periodontal patients screening.

The opportunity to interact not only with bacterial deposits, but also with morphological and histological characteristics of gingival tissues [4, 5, 25], provides a valuable opportunity for the clinician. Many clinical and biological concepts support the relevance of correctly stimulated interproximal tissues. It is now recognized that epithelial cells are not passive bystanders in periodontal tissues but are rather metabolically active and capable of reacting to external stimuli by synthesizing a number of cytokines, adhesion molecules, growth factors, and enzymes. Furthermore, epithelial cells generate a family of potent antimicrobial peptides that provide protection against infection. These peptides, called defensins, appear to work in concert with other host defense mechanisms to struggle with multiple microbial species and form a first line of host defense [26]. These peptides are localized in the differentiated layers of gingival epithelium and are absent in basal cells. Expression of these peptides is the strongest in the external portion of the tissue, particularly at the gingival margin [27]. Modification of periodontal tissues induced by “hygiene therapy” can increase the differentiated layers [4, 5, 26], possibly through the increased production of defensin peptides. Following such therapy, the stimulated tissue may be more resistant to infection.

## 5. Conclusions

IPI can be considered a reliable and practical index for use by dental hygienists, periodontists, and general dentists. During initial preparation or maintenance, the clinician can use IPI to verify tissue modification as a reliable indicator of patients compliance with recommended hygiene protocols.

In addition to these potential benefits, our clinical experience has indicated that many other practical advantages result from a properly stimulated periodontium. Considering the surgical aspects, smoothened inflammation, and edema reduce bleeding during incision, improving operator visibility; a better incision design is also achieved as the blade moves in more stable tissues. Consistency of tissues helps to raise flaps without leaving abundant soft-tissue remnants. Moreover, the increased tonicity of the gingiva and specifically of the interdental tissues allows the periodontist to better manage and suture the tissues.

Finally, we believe that this index can have a practical use not only for the clinician, but for the patient hygienic motivation too. Watching in a mirror, while the clinician obtains the IPI scores, the opportunely instructed patient can see the gingival response to the probe pressure, perceiving the sites that are not adequately stimulated or remain clearly inflamed [28].

In conclusion, patient compliance to oral hygiene instructions is essential in periodontal therapy and the new IPI index can be a practical and intuitive tool to check and reinforce this important aspect.

## Figures and Tables

**Table 1:** Interdental pressure index (IPI).

IPI		
Score	Clinical signs	Judgment
Positive	(i) Consistency: spongy and unstable (ii) Color: relevant ischemia (iii) Bleeding	Tissues are not correctly stimulated

Negative	(i) Consistency: tonic and firm (ii) Color: slight ischemia or stable	Tissues are correctly stimulated

**Table 2:** Histological parameters and corresponding scores.

Parameter	Score 1	Score 2	Score 3
Fibrosis	Mild	Moderate	Severe
Hyperkeratosis	Mild	Moderate	Severe
Amount of blood vessels	Large	Medium	Small
Inflammation	Severe	Moderate	Mild
Epithelial hyperplasia	Severe	Moderate	Mild

**Table 3:** Validation criteria results between gingival index (GI) and interdental pressure index (IPI) with different cutoffs.

GI cutoff	Sensitivity	Specificity	Positive predictive value	Negative predictive value
GI = 0 versus GI = 1 + 2 + 3	0.55	1.00	1.00	0.36
GI = 0 + 1 versus GI = 2 + 3	0.82	0.86	0.82	0.86
GI = 3 versus GI = 0 + 1 + 2	1.00	0.64	0.27	1.00

**Table 4:** Distribution of interproximal areas on the basis of pocket bleeding index (PBI), gingival index (GI), and clinical interdental pressure index (IPI) determinations.

	PBI+ *n* (%)	PBI− *n* (%)	GI = 0 or 1 *n* (%)	GI = 2 or 3 *n* (%)
IPI+	11 (100)	—	2 (18)	9 (82)
IPI−	3 (21)	11 (79)	12 (86)	2 (14)
Fisher's exact test	*P* = 0.0001		*P* = 0.001	

**Table 5:** Distribution of histological grades and clinical interdental pressure index (IPI) determinations in interproximal specimens.

	Grade I Acute active form	Grade II Intermediate form	Grade III Chronic inactive form
IPI+	12	3	0
IPI−	0	3	12
Chi-square	*P* = 0.0001		

## References

[1] Lang NP (1983). Indications and rationale for non-surgical periodontal therapy. *International Dental Journal*.

[2] Greenstein G (1992). Periodontal response to mechanical non-surgical therapy: a review. *Journal of Periodontology*.

[3] Bouwsma O, Caton J, Polson A, Espeland M (1988). Effect of personal oral hygiene on bleeding interdental gingiva. Histologic changes. *Journal of Periodontology*.

[4] Cantor MT, Stahl SS (1965). The effects of various interdental stimulators upon the keratinization of the interdental col. *Periodontics*.

[5] Checchi L, Biagini G, Zucchini C, de Luca M (1991). Clinical and morphologic response to interdental brushing therapy. *Quintessence International*.

[6] Amato R, Caton J, Polson A, Espeland M (1986). Interproximal gingival inflammation related to the conversion of a bleeding to a nonbleeding state. *Journal of Periodontology*.

[7] Caton J, Bouwsma O, Polson A, Espeland M (1989). Effects of personal oral hygiene and subgingival scaling on bleeding interdental gingiva. *Journal of Periodontology*.

[8] Saxer UP, Mühlemann HR (1975). Motivation and education. *Schweiz Monatsschr Zahnheilkd*.

[9] Abrams K, Caton J, Polson A (1984). Histologic comparisons of interproximal gingival tissues related to the presence or absence of bleeding. *Journal of Periodontology*.

[10] Graves RC, Disney JA, Stamm JW (1989). Comparative effectiveness of flossing and brushing in reducing interproximal bleeding. *Journal of Periodontology*.

[11] Armitage GC (1999). Development of a classification system for periodontal diseases and conditions. *Annals of Periodontology*.

[12] Saklad M (1940). Grading of patients for surgical procedures. *Anesthesiology*.

[13] Löe H, Silness J (1963). Periodontal disease in pregnancy. Prevalence and severity. *Acta Odontologica Scandinavica*.

[14] van der Velden U (1979). Probing force and the relationship of the probe tip to the periodontal tissues. *Journal of Clinical Periodontology*.

[15] Landis JR, Koch GG (1977). The measurement of observer agreement for categorical data. *Biometrics*.

[16] Green JC, Vermillion JR (1960). Oral hygiene index: a method for classifying oral status. *Jouranl of American Dental Association*.

[17] Löe H (1967). The gingival index, the plaque index and the retention index systems. *Journal of Periodontology*.

[18] Silberman SL, le Jeune RC, Serio FG, Devidas M, Davidson L, Vernon K (1998). A method for determining patient oral care skills: the University of Mississippi oral hygiene index. *Journal of Periodontology*.

[19] American Academy of Periodontology (1992). *Periodontal Screening and Recording System Training Manual*.

[20] Newbrun E (1996). Indices to measure gingival bleeding. *Journal of Periodontology*.

[21] Dababneh RH, Khouri AT, Smith RG, Addy M (2002). A new method of plaque scoring: a laboratory comparison with other plaque indices. *Journal of Clinical Periodontology*.

[22] Dhingra K, Vandana KL (2011). Indices for measuring periodontitis: a literature review. *International Dental Journal*.

[23] Lang NP, Attstrom R, Löe H, Lang NP, Attstrom R, Löe H (1998). Commonly used indices to assess oral hygiene and gingival and periodontal health and diseases. *Proceedings of the European Workshop on Mechanical Plaque Control*.

[24] Trombelli L, Tatakis DN, Scapoli C, Bottega S, Orlandini E, Tosi M (2004). Modulation of clinical expression of plaque-induced gingivitis—II. Identification of “high-responder” and “low-Responder” subjects. *Journal of Clinical Periodontology*.

[25] Caton JG, Blieden TM, Lowenguth RA (1993). Comparison between mechanical cleaning and an antimicrobial rinse for the treatment and prevention of interdental gingivitis. *Journal of Clinical Periodontology*.

[26] Bartold PM, Walsh LJ, Narayanan AS (2000). Molecular and cell biology of the gingiva. *Periodontology 2000*.

[27] Dale BA (2002). Periodontal epithelium: a newly recognized role in health and disease. *Periodontology 2000*.

[28] Rateitschak KH, Rateitschak EM, Wolf HF, Hassell TM (1985). *Color Atlas of Periodontology*.

